# Field Testing Multi-Parametric Wearable Technologies for Wildfire Firefighting Applications

**DOI:** 10.3390/s25103066

**Published:** 2025-05-13

**Authors:** Mariangela Pinnelli, Stefano Marsella, Fabio Tossut, Emiliano Schena, Roberto Setola, Carlo Massaroni

**Affiliations:** 1 Unit of Measurements and Biomedical Instrumentation, Departmental Faculty of Engineering, Università Campus Bio-Medico di Roma, Via Alvaro del Portillo, 21, 00128 Rome, Italy; mariangela.pinnelli@unicampus.it (M.P.); e.schena@unicampus.it (E.S.); 2 Unit of Automatic Control, Departmental Faculty of Engineering, Università Campus Bio-Medico di Roma, Via Alvaro del Portillo, 21, 00128 Rome, Italy; r.setola@unicampus.it; 3 Italian Firefighting Corp, Piazza del Viminale, 1, 00184 Rome, Italy; stefano.marsella@vigilfuoco.it (S.M.); fabio.tossut@vigilfuoco.it (F.T.); 4 Fondazione Policlinico Campus Bio-Medico di Roma, 00128 Rome, Italy

**Keywords:** wearable technology, physical monitoring, physiological monitoring, live fire training, wildland firefighting

## Abstract

In response to the escalating complexity and frequency of wildland fires, this study investigates the feasibility of using wearable devices for real-time monitoring of cardiac, respiratory, physical, and environmental parameters during live wildfire suppression tasks. Data were collected from twelve male firefighters (FFs) from the Italian National Fire Corp during a simulated protocol, including rest, running, and active fire suppression phases. Physiological and physical metrics such as heart rate (HR), heart rate variability (HRV), respiratory frequency (f_R_) and physical activity levels were extracted using chest straps. The protocol designed to mimic real-world firefighting scenarios revealed significant cardiovascular and respiratory strain, with *HR* often exceeding 85% of age-predicted maxima and sustained elevations in high-stress roles. Recovery phases highlighted variability in physiological responses, with reduced HRV indicating heightened autonomic stress. Additionally, physical activity analysis showed task-dependent intensity variations, with debris management roles exhibiting consistently high exertion levels. These findings demonstrate the relevance of wearable technology for real-time monitoring, providing an accurate analysis of key metrics to offer a comprehensive overview of work-rest cycles, informing role-specific training and operational strategies.

## 1. Introduction

Wildland-urban interface fire suppression is an arduous task that places substantial physical and psychological demands on firefighters (FFs). Due to global warming and increasing population density, the frequency and intensity of wildfires have escalated significantly, leading to profound adverse effects on the environment, economy, and society [1]. In 2024, according to the European Forest Fire Information System (EFFIS), Italy alone witnessed over 600 wildfires in the first 7 months, burning approximately 220 km^2^ of land [2].

Unlike urban firefighting, rural or wildland firefighting requires combating fires across unpredictable terrains and often under rapidly changing and extreme environmental conditions [3]. Traditionally, the majority of research into firefighting tactics has been conducted under laboratory-based controlled conditions simulating urban fire environments [4]. While various simulation models have demonstrated effectiveness in replicating fire behaviors in theoretical scenarios, and provide accessible web-based simulation platforms for broader use, they still fall short in fully capturing the real-world complexities faced during actual wildfire events characterized by high unpredictability [5,6,7,8] and overlook the subjective and physiological experiences of FFs themselves, who face unique and varied challenges in the field [9].

In fact, recent data from the National Fire Protection Association (NFPA) in 2023 show that overexertion and stress were responsible for more than half (48 deaths, 54%) of FFs fatalities, predominantly due to sudden cardiac events. The report from 2022 reveals that out of a total of 94 FFs fatalities in US, the 40% of whom were related only to rural fire departments, were due to stress and overexertion [10,11].

Crucially, the physiological load on FFs is shaped by a complex interplay of work practices, which include navigating steep terrains, enduring high environmental temperatures while inhaling smoke, and handling heavy personal protective equipment (PPE) [12]. This equipment, typically comprising turnout jackets and pants, a self-contained breathing apparatus (SCBA), gloves, boots, a face mask, and personal tools, can weigh between 5 and 20 kg [13]. Adding to these challenges are the tactical approaches employed during wildfire suppression, which can be either direct or indirect. Direct attacks involve attempting to extinguish flames closely, whereas indirect attacks are conducted from a greater distance, up to 100 m, focusing on isolating the burning fuel from unburned vegetation by creating fire lines [14]. However, the differential impact of these strategies on FFs’ physiological responses remains underexplored.

Thus, firefighting requires a high level of cardiorespiratory fitness to perform operational tasks safely and effectively [15]. This intense environment not only challenges the physical endurance of FFs but also their decision-making abilities under stress. Ongoing physiological evaluation is key in enhancing safety protocols and optimizing work-rest intervals [16]. In light of these challenges, monitoring specific physiological parameters becomes crucial for ensuring the health and efficiency of FFs during operations. Key metrics such as cardiac and respiratory frequencies are particularly vital due to their roles in reflecting the immediate stress responses and overall cardiovascular load.

Upon arriving at a fire scene, heart rate (HR) may already be elevated as a stress reaction to the initial fire alarm and continues to rise throughout firefighting tasks, often reaching or exceeding the predicted maximal [15,17]. For instance, during simulated high-rise firefighting, *HR* has been shown to be significantly higher during fire suppression tasks compared to other duties, underscoring the intense physiological demands of these activities [18]. Moreover, the physiological burden from firefighting can lead to incomplete recovery, evidenced by persistently elevated *HR* and lactate levels even after periods of rest [19].

To effectively monitor and manage this strain, the American College of Sports Medicine (ACSM) provides guidelines for training targets based on *HR* measurements. According to the ACSM, maintaining *HR* at 85% of the age-dependent maximum (*HR_max_*) during intense activities helps gauge the severity of exertion and categorize activities into ‘hard’ or ‘very hard’ levels [20,21]. Beyond just monitoring peak exercise levels, Heart Rate Recovery (HRR)—the rate at which the *HR* returns to normal after exercise—serves as a key indicator of autonomic nervous system efficiency and overall cardiovascular recovery. A slower HRR can signal underlying risks such as potential myocardial injury, underscoring the importance of adequate recovery time and protocols [19,22].

To further assess autonomic stress response and the cardiovascular regulation of heart rhythm, Heart Rate Variability (HRV) is studied. HRV has been applied to detect stress in workers exposed to extreme heat and provide decision support [23]: reduced HRV is associated with impaired cognitive and physical performance, especially under job-related stressors, thus could impact FFs’ decision-making ability [24,25]. Among the various metrics available, the time-domain measure of HRV, particularly the root mean square of the successive differences (RMSSD) between adjacent *RR* intervals, is of great relevance. This metric represents a measure of beat-to-beat variability (deriving from R peaks of the ECG signal), reflects short-term variability and parasympathetic nervous system activity and offers a focused evaluation of vagal tone, being less influenced by breathing rate [19,26]. For FFs, who operate under extreme stress and need to maintain high alertness, *RMSSD* provides insight into their ability to quickly recover after intense activities. A higher *RMSSD* indicates a more effective autonomic nervous system response, particularly in its parasympathetic component, which is responsible for body relaxation and recovery. This is crucial for FFs as it directly affects their ability to make rapid, clear-headed decisions during emergencies.

Similarly, research indicates that respiratory frequency (f_R_) is acutely sensitive to environmental stressors such as heat, cold, and hypoxia, with significant increases during both rest and intense physical activity, closely correlating with body temperature [15,27]. This sensitivity underscores the importance of monitoring f_R_ to identify FFs at risk of heat strain, especially given the elevated respiratory rates observed during firefighting. For instance, studies indicate that f_R_ can reach as high as 80 respirations per minute (rpm) during intense activities, compared to normal resting rates of around 40 rpm, highlighting the physiological extremes experienced [28]. Moreover, the presence of abnormal respiratory health indicators, such as spirometry and impulse oscillometry readings, in FFs underscores the need for ongoing respiratory health surveillance [29]. The responsiveness of f_R_ to task conditions is also noteworthy, particularly in the context of firefighting. Associated with exercise-induced fatigue, such as muscle fatigue, f_R_ reacts quickly to changes in task conditions: for instance, at the onset and offset of physical exertion. This rapid response makes f_R_ a valuable indicator for adjusting tactics in real-time, enhancing not only the safety but also the effectiveness of firefighting operations. By accurately assessing f_R_, strategies can be tailored to maximize performance and reduce the risk of fatigue during critical tasks [30]. Additionally, f_R_ is also amenable to modifications through both direct and indirect interventions. For instance, techniques that focus on mindful attention to breathing can adjust f_R_ and have been shown to transiently enhance mood and reduce emotional reactivity during high-stress scenarios as those of FFs [31]. This adaptability of f_R_ makes it an essential metric for interventions designed to manage stress and improve overall outcomes in physically demanding activities.

Thus, thanks to technological advancements in wearable sensors, it is now more feasible to gather physiological data noninvasively and in real time [32,33]. Such devices must offer wireless connectivity, long battery life, and the durability needed to withstand the extreme and dynamic conditions of wildfire suppression. Despite numerous studies exploring wearable sensors under PPE, there is still a need for improvements to comprehensively capture all necessary parameters for monitoring FFs’ physiological extremes in real-world conditions [32]. The hazardous environments encountered during real fire activities limit the ability to easily test scenarios to improve the safety and effectiveness of interventions or to decrease physiological strain. Although most of the research on FFs’ physiological responses has been conducted in laboratory conditions or through controlled simulations, only a limited number of studies have investigated physiological parameters during real firefighting operations [15]. The breadth of research analyzing physiological variables and physical parameters underscores the utility of sensor-based systems in enhancing FFs safety without disrupting operational procedures. Systems such as portable metabolic analyzers, *HR* monitors, ingestible temperature pills, and skin temperature patches have proven effective for monitoring physiological aspects [12,15,27,34,35]. Within this context, studies vary in their approach to validation and application: refs. [21,36] tested wearable commercial armbands and suits via simulations focusing on simulated firefighting tasks; refs. [37,38] included laboratory validations and subsequent field trials to confirm the reliability and effectiveness of sensor-based monitoring in active firefighting scenarios; meanwhile, ref. [39] developed a smart T-shirt for monitoring thermal stress but did not advance to real-world testing, underscoring a disconnect between prototype development and practical deployment. This discrepancy emphasizes the need for comprehensive field testing to verify the operational readiness and effectiveness of new technologies in actual unpredictable firefighting wildfire scenarios, comprising diverse firefighting tactics.

Therefore, our study aims to rigorously test the feasibility of non-intrusive wearable devices in challenging live fire conditions. Specifically, we tested a commercial wearable multi-parametric device worn by all participants to collect cardiac, respiratory, and acceleration data. To complement these physiological measurements with environmental context, we also integrated an external datalogger positioned in the chest pocket to monitor environmental temperature during the wildfire simulation. By deploying advanced wearable technology to collect real-time data from FFs, this approach allows for an in-depth examination of the physical strain FFs endure across various phases of firefighting, including rapid deployment, active suppression, and recovery. Through the analysis of specific physiological (cardiac and respiratory responses), physical (activity levels), and environmental (external temperature) conditions of FFs during wildfires suppression, this study aims to evaluate the practical applicability of these technologies directly in the field. This approach will provide a detailed assessment of the physiological strain endured by FFs, enabling the proposal of modifications to work-rest cycles and operational tactics in training activities.

## 2. Materials and Methods

Physiological and physical data were collected from 12 active male FFs from the National Fire Corps (CNVVF) of Rome, who were divided into two teams (Team 1 and Team 2), each composed of six FFs. The teams included a total of three instructors (54 ± 1 year, 1.74 ± 0.07 m, 76.3 ± 10.6 kg) and nine trainees, whose ages ranged from 21 to 43 years (32 ± 7 year, 1.80 ± 0.07 m, 75.8 ± 6.9 kg). All participants were equipped with standard firefighting gear which includes a fireproof overall (65% viscose, 30% nomex, and 5% evlar), helmet, gloves, a face-neck shroud, and midcalf leather boots. Notably, only Team 1 was equipped with FFP3 masks during the data collection phases. Additionally, all of them carried different tools (3 kg–20 kg) to perform their job. Moreover, instructors were specifically outfitted with a Multi-functional Operational Helmet (MCO).

**Figure 1:**
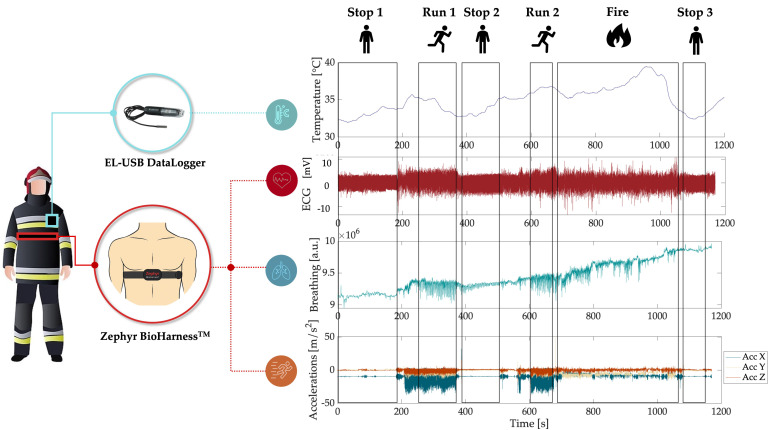
Wearable monitoring systems placement on a FF during the protocol. From top to bottom, the plots, respectively, show temperature (measured via EL-USB Datalogger), and ECG, respiratory, and acceleration signals along the X, Y, and Z axes (from the Zephyr BioHarness^TM^ chest strap) over approximately 1200 s of testing. The distinct phases of the exercise are highlighted by the outlined boxes.

All tests were conducted at the Multifunctional Training Center in Montelibretti (Rome), the FFs’ training center equipped with multiple practical simulation areas, including those for wildfire scenarios. None of the participants had a history of cardiopulmonary or intestinal diseases, and no musculoskeletal disorders were reported. All participants provided written informed consent before data collection commenced. Data were acquired using a commercial thoracic band, the Zephyr BioHarness^TM^ 3.0 (by Medtronic Inc., Minneapolis, MN, USA), which was worn by all participants to monitor cardiac and respiratory parameters. The BioHarness is worn using an elastic chest strap with an adjustable tensioning system and buckle. No adhesive elements are used. For optimal signal acquisition, the strap is placed snugly around the thorax, immediately below the pectoral region, with the central module positioned under the left armpit. The device is worn directly in contact with the skin (as illustrated in Figure 1), under the flame-resistant undershirt and the protective jacket, as part of the standard layering of PPE. This configuration ensures stable positioning and optimal contact of both the ECG electrodes and the respiratory inductive sensor, as recommended by the manufacturer. The system includes a single-lead ECG sensor with two dry textile electrodes, which operate by detecting the bioelectrical activity of the heart through direct contact with the skin. To ensure optimal conductivity, the electrodes must be slightly moistened before each use, as specified in the user manual. The chest strap also embeds a strain-based sensor for respiratory signal acquisition (sampling rate: 25 Hz) and a 3-axis accelerometer (sampling rate: 100 Hz) for movement analysis. The ECG is sampled at 250 Hz via the textile electrodes.

Moreover, external temperature was recorded using the EL-USB DataLogger 7.7 (by Lascar Electronics, Whiteparish, Wiltshire, UK), a standalone device that logs over 32,000 readings from a thermistor probe with a measurement range of −40 to 125 °C with an accuracy of ±0.5 °C within the −5 °C to 40 °C range and ±1.0 °C from 40 °C to 125 °C. The logger consists of a compact main unit connected to a cabled thermistor. For the experiment, the main body of the device was placed inside the jacket pocket, while the thermistor cable was routed externally, with the sensor tip positioned outside the pocket to directly measure the environmental temperature. Although the datalogger was not part of the physiological monitoring system under test, it was integrated to provide a reliable estimation of the external temperature experienced by the FFs during wildfire suppression. This allowed us to contextualize the physiological data and verify the thermal environment in which the wearable system operated. The collected equipment was recharged and refreshed, and data were downloaded from memory cards of the straps.

**Figure 2:**
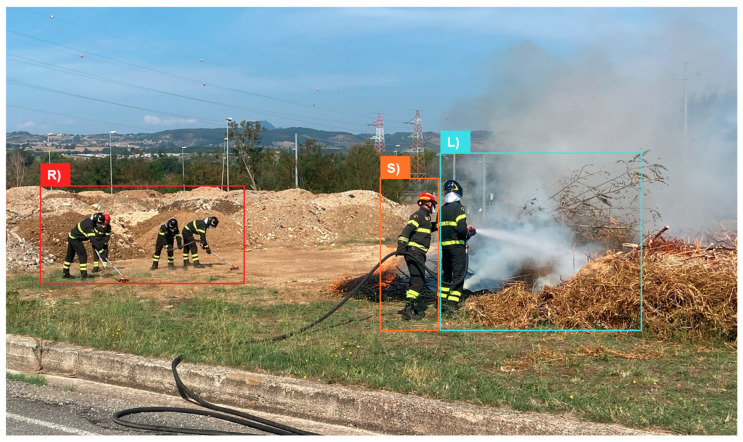
FFs of Team 1 performing different tasks during fire suppression phase. In area R (red box), FFs are clearing the ground using rakes. In area S (orange box), a FF is providing hose support while in area L (light blue box), a FF is actively extinguishing a controlled fire with the hose.

The study followed a controlled simulation protocol (Figure 1), meticulously designed to replicate standard recovery and action sequences typical in firefighting scenarios. The protocol included:

- ‘Stop 1’: 3 min of baseline data collection to establish physiological norms while the participants were at rest.
- ‘Run 1’: 2 min of running to elevate physiological stress
- ‘Stop 2’: 2 min of static rest to observe recovery patterns.
- ‘Run 2’: 1 additional min of running simulating an approach towards a fire.
- ‘Fire’ (Figure 2): 5 min of fire suppression, during which the team was divided into three functional roles: handling the hose for direct fire suppression (‘L1’ for Team 1, ‘L2’and ‘L3’ for Team 2), providing hose support (‘S1’ for Team 1 and ‘S2’ for Team 2), and using rakes to manage surrounding debris (‘R1’,’R2’,’R3’,’R4’, in Team 1, ‘R5’ and ‘R6’ for Team 2).
- ‘Stop 3’: 1 min of data collection during the post-suppression recovery phase to assess physiological return to baseline.

This protocol was designed to provide a comprehensive assessment of the physiological impacts of various firefighting tasks, as well as the environmental conditions faced (Figure 2).

### 2.1. Data Processing

Raw data collected from the chest strap were processed in the MATLAB 2024a environment. Subjects ‘L1’ and ‘R5’ were excluded from the analysis due to degraded measurement quality (e.g., inadequate data resolution, low signal-to-noise ratio) in the chest strap module, thus allowing the analysis of 83.3% of data.

#### 2.1.1. Physiological Signals Processing

The processing of physiological signals for both ECG and breathing involved precise filtering techniques to ensure accurate assessments of *HR* and f_R_ from data captured via sensors integrated into the FFs’ chest straps during these particular tasks.

**Figure 3:**
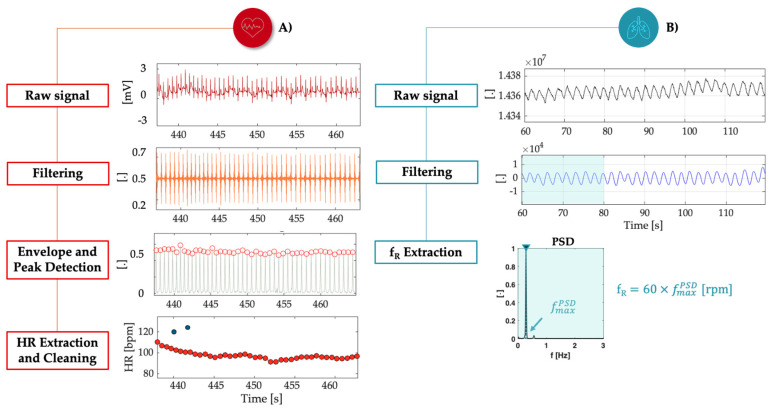
(**A**) Segment of raw ECG signal collected from the chest strap device of subject ‘S3’ in phase ‘Stop 2’, filtered ECG, envelope of the filtered signal and detected beats in red marks, followed by the *HR* estimated from the peaks after cleaning the extra beats in blue marks. (**B**) Breathing raw signal collected by the strap, filtering and processing of a signal segment using the Welch method, the selection of a 20 s sliding window, and the extraction of the maximum frequency and subsequent f_R_.

Starting from the cardiac responses, for each of the 6 FFs in both Team 1 and Team 2, ECG signals were collected using chest strap electrodes and processed to address the inherent noise and artifacts (Figure 3A). The preprocessing began with a first-order Butterworth bandpass filter set within the 20–30 Hz range. This frequency band was carefully selected to remove unwanted noise while preserving the essential components of the ECG signal, particularly the QRS complexes, which represent individual heartbeats. To further enhance the identification of R-peaks, the signal envelope was computed, allowing for the precise localization of these critical features in the time series. A subsequent data cleaning process was applied, in which outliers were removed by comparing the extracted *HR* values with the preceding and subsequent *HR* trends within a 10 s window. Any value deviating significantly from this trend was defined as an outlier and excluded from further analysis [40]. Once the R-peaks were identified, *HR* values were calculated based on the time intervals between consecutive peaks, known as *RR* intervals, defined as the time difference between successive R-peaks (Equation (1)). The *RR* interval served as the foundation for *HR* computation expressed in beats per minute (bpm) (Equation (2)).

(1) $${RR}_{i}=t_{QRS}\left(i+1\right)-t_{QRS}\left(i\right)$$

(2) $$HR(t)=\frac{60}{RR\;(t)}$$

However, the dynamic and extreme conditions under which the data were collected introduced artifacts and ectopic beats into the signal, necessitating a data cleaning process to ensure the accuracy of *HR* estimations. To refine the *RR* interval series, an iterative algorithm was applied to detect and remove anomalous beats, often referred to as extra beats. The detection process involved calculating the difference between consecutive *RR* intervals (Equation (3)) and comparing these differences to a predefined threshold (thr) of 50 ms, as proved physiologically (Equation (4)). Intervals exceeding the thr were classified as anomalous and removed from the time series.

(3) $$∆{RR}_{i}={RR}_{i+1}-{RR}_{i}$$

(4) $${|∆RR}_{i}|>thr$$

After each iteration, the number of removed beats and their percentage relative to the total initial *RR* intervals were calculated, ensuring a thorough data cleaning process. By the end of the process, the percentage of removed beats was computed to quantify the impact of artifacts on the original dataset (see Table 1). This final percentage highlights the algorithm’s effectiveness in identifying and removing unusable portions of the signal, even in cases with significant artifacts, such as for subject ‘R3’.

Similarly, the respiratory signals were handled with a comparable level of rigor to ensure reliability in f_R_ analysis. The breathing signals, collected from piezoresistive sensors integrated into the chest strap, were filtered using a first-order Butterworth filter with a frequency range of 0.1 Hz to 0.7 Hz, chosen to isolate the expected respiratory rates, ensuring the focus remained on the physiological breathing pattern of interest. However, the analysis faced significant challenges, as the signal was heavily compromised by motion artifacts, particularly during the dynamic phases of the protocol. Due to these limitations, a direct time-domain analysis proved unreliable for accurately capturing f_R_. To overcome this, a power spectral density (PSD) approach was employed to track the dominant frequency trends over time. This approach was chosen for its robustness against motion artifacts and has been validated in previous studies [27,35,41], allowing for consistent estimation of respiratory trends under dynamic conditions. The PSD was calculated using a 20 s window with a 1 s overlap, assuming a sampling frequency of 25 Hz for the breathing signal [42]. This allowed for the identification of the dominant respiratory frequency in each time window, which was then converted to rpm by multiplying the frequency by 60 (Figure 3B).

#### 2.1.2. Physical Data Processing

The analysis of physical parameters relies on data extracted from an accelerometer integrated within the chest strap module. The acceleration signals are processed by filtering between 0.1 Hz and 0.8 Hz on each of the triaxial accelerometer’s axes (x, y, z) to capture frequencies pertinent to human movement. This targeted filtering enhances the accuracy of movement analysis by eliminating irrelevant noise specific to each axis, such as gravity artifacts, thereby ensuring precise measurement of the FFs’ physical activities during various tasks [43].

After filtering, the physical activity level of FFs was quantified by means of the Vector Magnitude Unit (VMU) on each of the six phases of the protocol (Equation (5)). The VMU is derived by calculating the square root of the sum of the squares of the individual acceleration along the three axes. This value allows for a precise measurement of movement intensity:

(5) $$VMU=\sqrt{x^{2}+y^{2}+z^{2}}$$

The conversion from the acceleration value, expressed in m/s^2^, to VMU in gravities (g) is then accomplished under varying conditions of heat exposure.

## 3. Results

This section presents a comprehensive analysis of the data gathered during the wildfire suppression simulation, focusing on physiological, physical, and environmental metrics. The results are structured to first elaborate on the physiological responses of the FFs under varying conditions of the simulation protocol, followed by insights into their physical levels of activity and the surrounding environmental conditions.

### 3.1. Physiological Data

Following the extraction of HR, it was possible to analyze key metrics in each phase in detail, as defined in Section 1. The metrics of interest included *HR* ranges during each phase and maximum *HR* values (MHR) compared to the threshold defined by the ACSM along with their percentage deviation (%MHR) (Section 3.1.1), as well as HRR during the two stop phases after actions and HRV in the time domain, expressed as *RMSSD* (Section 3.1.2).

#### 3.1.1. Heart Rate Range and Maximum Measures

**Figure 4:**
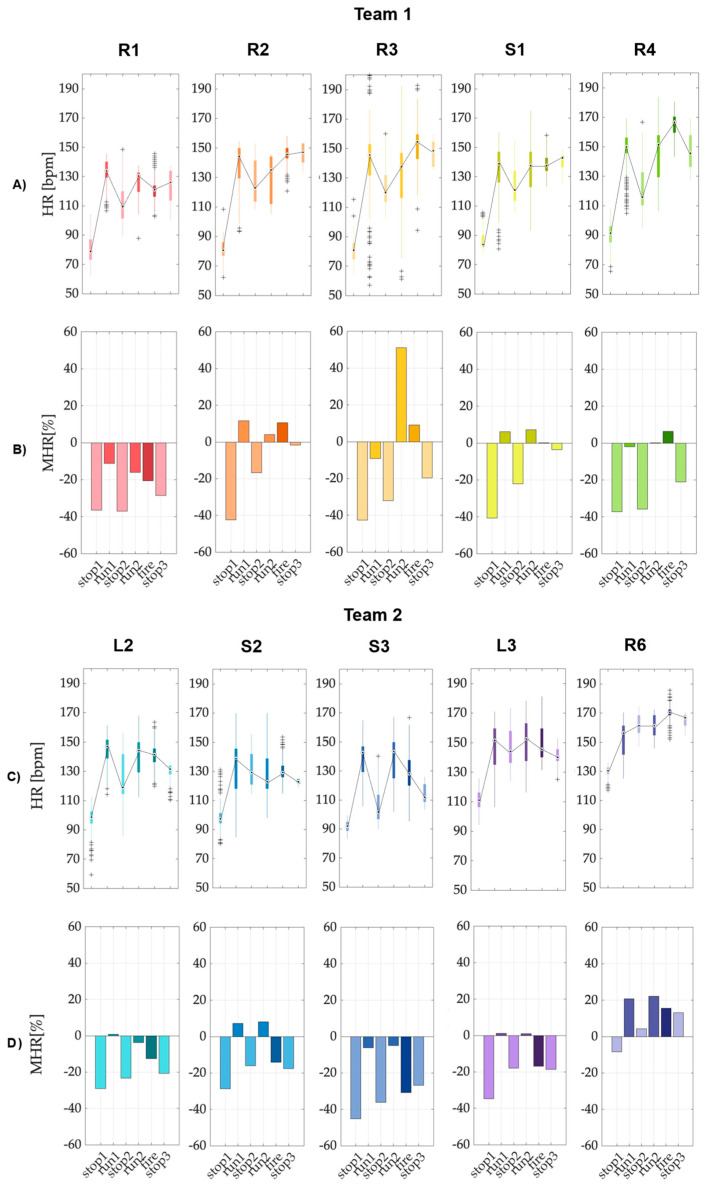
(**A**,**C**) Boxplots of *HR* values for each phase of each FFs (all subjects of each team, Team 1 and Team 2, are reported), where the central black dot indicates the median, and the bottom and top edges of the boxes indicate the 25th and 75th percentiles, respectively. The whiskers extend to the most extreme data points not considered outliers, and the outliers are plotted individually using the black ‘+’ markers. (**B**,**D**) Percentage of Maximum Heart Rate (%MHR) for each firefighting phase and subject. This chart displays how each participant’s *HR* compares to their age-adjusted 85% *HR_max_* during each phase of the protocol.

The *HR* range indicates the variability between the lowest and highest HRs recorded during the phases, offering insights into cardiovascular responses during physical tasks. To effectively monitor physiological changes between static and dynamic phases of activity, boxplots are employed to visualize variations in *HR* (Figure 4A). The expected pattern typically shows a lower median *HR* during the recovery phases following intense activity. This trend reflects the physiological shift as the body transitions from a state of high exertion back to rest, allowing for a clearer assessment of cardio-vascular recovery dynamics. On the other hand, the MHR marks the peak cardiovascular effort exerted by an individual. As evidenced in Section 1, the ACSM guideline advises that intense training sessions should aim for 85% of the age-adjusted HR_max_ in bpm, defining:

(6) $${HR}_{max}=220-age\;[bpm]$$

In this way, exercise intensity is categorized as ‘hard’ or ‘very hard’ [20,21]. This standard is useful for determining whether the intensity of physical activity is appropriate relative to individual cardiovascular capabilities.

In the context of the study, the deviation of each participant’s MHR from their personal threshold is calculated during each phase of the exercise in percentual terms (%MHR, percent Maximum Heart Rate) (Figure 4B). A result below zero indicates *HR* within a safe range, suggesting that the physical exertion is manageable. Conversely, values exceeding zero may indicate a potential cardiac overload, signifying excessive physiological strain that warrants further analysis.

Particularly noteworthy is the observation that while it is rare for individuals to fail to reach 85% of their *HR_max_* during stress testing, significantly exceeding this threshold during actual activities requires careful evaluation. Achieving the 85% *HR_max_* threshold during activities such as running or fire drills is considered ideal, as it reflects an optimal level of exertion. However, failure to reach this threshold during stress testing is deemed abnormal and may indicate underlying cardiovascular issues that warrant further investigation [22].

#### 3.1.2. Heart Rate Recovery and Heart Rate Variability

In the recovery phases ‘Stop 2’ and ‘Stop 3’, following the more intense activity phases ‘Run 1’ and ‘Run 2’ and ‘Fire’, HRR was evaluated for each subject in both teams.

**Figure 5:**
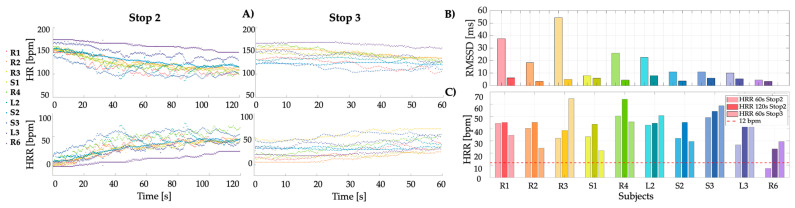
(**A**) *HR* changes over time in the first row, highlighting in the second row the HRR from the peak rates observed during the intense activities of Run 1 and Run 2 + Fire. (**B**) HRV values for each FF capturing data from two key rest phases, ‘Stop 1’ and ‘Stop 3’, at the beginning and the end of the protocol. For each subject, two bars are shown: the lighter shade indicates ‘Stop 1’ and the darker shade indicates ‘Stop 3’. (**C**) HRR values corresponding to 60 s and 120 s in ‘Stop 2’ and ‘Stop 3’ phases. For each subject, three bars are shown: the first and third share the same color, representing the first 60 s of ‘Stop 2’ and ‘Stop 3’; the middle bar (120 s) is shown in a different tone to highlight the later response.

Specifically, the *HR* trend over time during these intervals was analyzed, where a decreasing pattern associated with recovery was expected: the decline in *HR* during recovery is principally due to a reactivation of the parasympathetic nervous system, mostly in the early recovery period. In contrast, the HRR, calculated by comparing each HR value of the resting phases with the maximum *HR* reached at the end of the previous intense exercise phase (HR_0_), was expected to show an increasing pattern (Figure 5A). From these curves, values of HRR at 60 s epochs were extracted to evaluate the recovery progress at each minute. In the first recovery phase, ‘Stop 2’, two values were obtained, HRR_60_ and HRR_120_, while in the final recovery phase, ‘Stop 3’, only HRR_60_ was recorded.

HRR values after 1 and 2 min were saved and compared to a 12-bpm threshold (defined for upright position), which, if exceeded, indicates good health status during intense activities (Figure 5C): in fact, abnormal HRR is defined as the failure to decrease *HR* > 12 bpm per min during the first minute after peak exercise [22]. To further assess autonomic stress response, among the various metrics available, the time-domain measure of HRV, particularly the root mean square of the successive differences (*RMSSD*) between adjacent *RR* intervals, is of great relevance. This metric represents a measure of beat-to-beat variability (deriving from R peaks of the ECG signal), reflects short-term variability and parasympathetic nervous system activity, and offers a focused evaluation of vagal tone, being less influenced by breathing.

Supposing the *RR* interval time series includes N successive beat intervals, the *RMSSD* is defined as:

(7) $$RMSSD=\sqrt{\frac{1}{N-1}\sum_{n=1}^{N-1}{\left({RR}_{n+1}-{RR}_{n}\right)}^{2}},$$

where *RR_n_* denotes the value of n-th *RR* interval. Typically, during intense tasks, *RMSSD* values are expected to decrease, reflecting heightened sympathetic nervous system activity as the body responds to physical demands and stress. Conversely, higher *RMSSD* values are observed during periods of rest or less intense activities, indicating dominant parasympathetic activity that facilitates recovery. It is important to note that the normal range for adult HRV, as measured by the *RMSSD* parameter, can extend from below 20 to over 70 ms at rest [44]. However, HRV can vary significantly among individuals, influenced by factors like age, gender, fitness, and overall health. This variability underscores the need for personalized approaches in monitoring and training based on individual physiological responses. As firefighting tasks escalate in intensity, especially after significant physical exertion and high temperatures, a clear pattern in HRV is expected. Initially, in a relaxed state before engaging in physical tasks, a higher HRV (or high variability) is expected, showcasing a ready and responsive physiological state. Post-exertion, a reduced HRV (or low variability) typically follows, reflecting the body’s acute response to stress and physical demands [19]. This variation can be most accurately monitored during static phases—like ‘Stop 1’ and ‘Stop 3’—where *HR* signals are cleaner and less susceptible to movement artifacts (almost no outlier removal) (Figure 5B).

#### 3.1.3. Respiratory Data

Considering the intensity of actions, the f_R_ indicators were deeply analyzed in the recovery phases because f_R_ provided valuable insights into the physiological state of FFs under stress. Utilizing extracted data, the average rates for each subject were estimated, enabling the assessment of potential distress or relaxation states during these periods. This comprehensive respiratory data analysis, presented in Table 2, summarizes the observed mean f_R_, providing a broad view of the physiological extremes encountered by FFs during the study.

This analysis supports findings from previous research indicating that f_R_ is highly sensitive to environmental stressors such as heat, cold, and hypoxia. These conditions often cause f_R_ to escalate during intense physical activities and even during rest, reflecting the body’s efforts to compensate for increased metabolic demand and to maintain adequate oxygen supply.

Typically, respiratory rates can vary within the following ranges:

- Bradypnea, characterized by a slow breathing rate, is typically less than 12 rpm and may indicate a state of relaxation or, in some cases, respiratory suppression.
- Eupnea, or normal, un-labored breathing, from 12 to 20 rpm, reflects a stable and comfortable state of respiration.
- Tachypnea, which is an elevated respiratory rate exceeding 20 rpm, often occurs in response to stress or increased physiological demand, serving as a mechanism to enhance oxygen intake and carbon dioxide expulsion.

In the recovery phases following intense exertion, a transition from tachypnea back toward eupnea is expected as the body recovers. Upon analyzing the mean f_R_ data collected at the beginning ‘Stop 1’ and end of the protocol ‘Stop 3’, it was observed that all subjects generally returned to a state of eupnea by the end of the simulation, with average values falling within the normal breathing range of 12 to 20 rpm. However, an exception was noted for subject S2 in Team 2, who exhibited f_R_ starting from 21 rpm and above even at the onset. This indicates that the subject entered the protocol in a state of mild tachypnea, which may suggest either pre-existing stress or an initial heightened physiological arousal.

### 3.2. Physical Data

The extraction of VMU data has enabled a comprehensive assessment of physical activity levels among FFs during the simulated tasks. By analyzing the VMU, which quantifies the total accelerative forces experienced by a FF, it is possible to evaluate the intensity of movement of the activities performed. The analysis employs thresholds sourced from the chest strap datasheet, to categorize activity levels into three distinct categories:

(i) Low Intensity (<0.2 g): activities registering under 0.2 g are considered low intensity, indicating minimal exertion like slow walking. This level is typically associated with tasks requiring little physical effort.
(ii) Moderate Intensity (0.2 g to 0.8 g): values within this range denote moderate activity levels, characteristic of standard intense walk\run activities. This intensity reflects the typical demands of firefighting operations that do not require maximal effort.
(iii) High Intensity (>0.8 g): readings above 0.8 g are categorized as high intensity, indicative of vigorous and sustained physical efforts. Such levels are usually required in emergency situations where rapid and intense actions are necessary.

**Figure 6:**
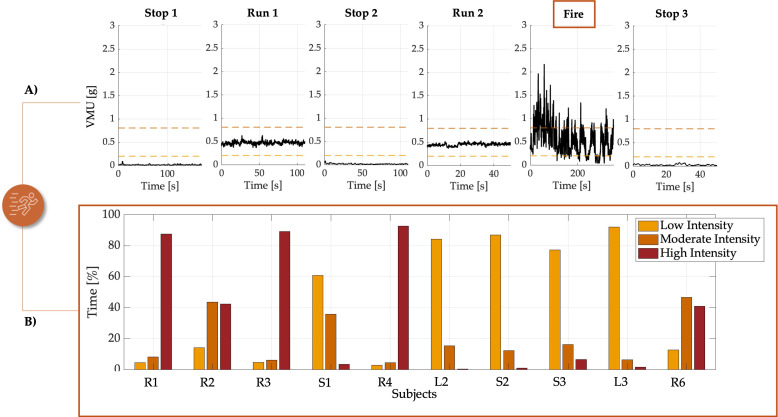
(**A**) VMU intensity levels (of subject R5 of Team 2) across all phases, showing the VMU signal over time (black line). The orange dashed lines represent threshold levels at 0.2 g (lower threshold) and 0.8 g (upper threshold). (**B**) Percentage of time spent in Low, Medium, and High intensity activities for all subjects.

The thresholds were consistently adhered to across various phases (see Figure 6A).

For example, during the ‘Stop 1’, ‘Stop 2’, and ‘Stop 3’ phases, all subjects-maintained activity levels under 0.2 g, showing coherence in low-intensity values across these resting phases. In contrast, during the running phases, all subjects exhibited moderate intensity levels, remaining within the 0.2 g to 0.8 g range. During the ‘Fire’ phase, activity intensity varied according to the specific task performed by each FF (see Figure 6B). During this phase, the highest maximal air temperatures were observed by the thermistors during the direct attack, with temperature peaks indicating the intense conditions faced by FFs. Specifically, the maximum temperatures reached were approximately 39.5 °C for Team 1 and 40.3 °C for Team 2. In terms of percentage distribution, FFs performing tasks that involved using rakes to manage surrounding debris (i.e., ‘R1’, ‘R2’, ‘R3’, and ‘R4’ in Team 1, and ‘R5’ and ‘R6’ in Team 2) maintained high-intensity levels (above 0.8 g) for a substantial portion of the time: three subjects remained in the high-intensity range for more than 85% of the time, while two others spent over 40% of their time at this intensity. On the other hand, those engaged in handling the hose for direct fire suppression (‘L1’ in Team 1, and ‘L2’ and ‘L3’ in Team 2) or providing hose support (‘S1’ in Team 1 and ‘S2’ in Team 2), which involved more static postures, exhibited lower intensity levels (below the high-intensity threshold) for over 60% of the time. This data-driven analysis highlights how activity intensity varies with specific firefighting tasks, providing insights into the physical demands associated with different roles.

## 4. Discussions and Conclusions

Wildland firefighting is a complex and demanding task that pushes the limits of both human endurance and technological capability. Traditionally, the majority of research into firefighting tactics and physiology has been confined to controlled laboratory settings or simulated environments [21,36,37,38] and rarely on live fire training scenarios [12,14]. This study bridges this gap by analyzing the interplay between physical demands, environmental conditions, and physiological responses in real-world wildfire suppression tasks during a live fire training scenario, in which FFs operated under real flames and field-like conditions within a structured simulation environment. Although conducted in a controlled training facility, the scenario closely replicated real wildfire suppression dynamics, providing a reliable context to assess the feasibility of wearable monitoring systems under operational stress. It validates the use of commercial wearable technologies under harsh conditions, enabling real-time monitoring and insights that inform targeted interventions to improve FFs safety and performance.

Our data collection demonstrates the importance of high-quality measurement devices, as degraded data from subjects ‘L1’ and ‘R5’ led to their exclusion, emphasizing the critical role of reliable hardware in ensuring robust analysis and maintaining data usability, with 83.3% of the dataset successfully analyzed. Additionally, the proposed ECG filtering and the data obtained from *HR* outliers’ detection pipeline demonstrate the good performance of the proposed algorithm in identifying and removing outliers across different stages and subjects. The algorithm shows robust cleaning performance during high-motion activities (e.g., running), where noise and artifacts are typically more prevalent. This suggests that the algorithm adapts well to dynamic conditions, ensuring accurate estimations even under non-ideal data collection environments. From cleaned data, key metrics such as %MHR, HRR, and HRV were extracted to evaluate cardiovascular stress and recovery. Analysis revealed that during intense activity, some subjects, such as ‘R3’ and ‘R6’, exceeded the ACSM thresholds by 40% and 17%, respectively, highlighting the strain imposed by firefighting tasks. The recovery phases further underscored the variability in physiological responses. While some subjects showed a reduction in *HR* during recovery, others, particularly those in physically demanding roles like debris management, exhibited sustained elevations, suggesting insufficient recovery or heightened stress. A notable decrease in HRV, particularly RMSSD, across all participants indicated the physiological toll of prolonged physical exertion, with certain individuals like ‘R6’ showing reduced variability from the outset, signaling a heightened susceptibility to stress and fatigue.

Respiratory monitoring, a typically underexplored parameter, was integrated into this analysis to provide a more comprehensive understanding of physiological stress. f_R_ was calculated using a PSD approach to account for motion artifacts, which were especially prevalent during dynamic tasks such as debris management. High f_R_ values were consistently observed during active phases, reflecting increased physical demands, while recovery phases demonstrated a return to normal respiratory rates. This analysis highlighted the importance of f_R_ as a marker of exercise-induced fatigue and its potential modulation through breathing-focused interventions.

Physical activity was further quantified using VMU to classify the intensity of various firefighting tasks. Debris management roles (‘R’ roles) showed significantly higher intensity levels, with over 40% of the ‘Fire’ phase spent in the high-intensity category, underscoring the continuous and vigorous nature of these tasks. Conversely, roles involving more static activities, such as hose handling, demonstrated lower overall intensity, emphasizing the diversity in physical demands among firefighting roles. Environmental factors, such as high temperatures, exacerbated fatigue, particularly during high-intensity tasks, highlighting the need for training and protocols tailored to specific roles and environmental stressors.

The findings of this study provide useful elements for identifying a data acquisition strategy to be used to evaluate the following matters: training programs could prepare FFs for the unique physical challenges of their assignments, such as endurance and strength training for debris management or positioning and stamina drills for hose handlers; safety protocols could be refined to incorporate real-time physiological monitoring, enabling adjustments in work-rest cycles to mitigate stress and optimize performance. Additionally, integrating environmental data such as humidity and other substance concentrations into real-world wildfire scenarios could better prepare FFs for the thermal and physiological stressors they face on the fire line. While this study was limited in sample size, it establishes a foundation for broader investigations that include a wider range of physiological and environmental variables. This approach not only enhances immediate safety measures but also paves the way for advancements in monitoring technologies and emergency response protocols, ultimately safeguarding FFs’ well-being in wildfire suppression.

## Figures and Tables

**Table 1:** *HR* outliers’ detection.

Subject	Extra Beats Removed (Percentage of Removal)					
	Stop 1	Run 1	Stop 2	Run 2	Fire	Stop 3
R1	52 (22%)	5 (2%)	0 (0%)	3 (2%)	0 (0%)	0 (0%)
R2	8 (3%)	7 (3%)	0 (0%)	0 (0%)	11 (1%)	0 (0%)
R3	90 (37%)	173 (60%)	3 (1%)	74 (60%)	247 (28%)	0 (0%)
S1	0 (0%)	86 (30%)	0 (0%)	61 (44%)	22 (3%)	0 (0%)
R4	35 (13%)	12 (4%)	3 (1%)	22 (15%)	29 (3%)	3 (2%)
L2	10 (3%)	110 (35%)	13 (5%)	23 (16%)	27 (3%)	0 (0%)
S2	88 (28%)	109 (41%)	36 (13%)	48 (37%)	100 (12%)	0 (0%)
S3	0 (0%)	39 (14%)	0 (0%)	33 (23%)	76 (10%)	3 (0%)
L3	0 (0%)	30 (10%)	7 (2%)	18 (12%)	10 (1%)	0 (0%)
R1	0 (0%)	0 (0%)	0 (0%)	1 (1%)	34 (0%)	0 (0%)

**Table 2:** f_R_ values expressed as mean ± standard deviation (std) calculated for all subjects over all the phases of the protocol.

	${f_{R}}_{mean\pm std}[rpm]$					
	Stop 1	Run 1	Stop 2	Run 2	Fire	Stop 3
R1	10.1 ± 1.7	22.1 ± 6.0	12.9 ± 2.0	13.7 ± 8.9	24.3 ± 8.1	11.8 ± 0.8
R2	12.5 ± 3.5	22.2 ± 6.6	19.3 ± 5.9	25.8 ± 11.4	20.6 ± 9.4	18.4 ± 5.9
R3	11.2 ± 2.2	15.3 ± 2.7	13.4 ± 1.5	11.8 ± 6.4	20.4 ± 4.2	15.1 ± 2.4
S1	15.7 ± 7.6	22.3 ± 1.8	15.8 ± 6.1	24.0 ± 1.4	15.5 ± 6.9	10.7 ± 2.3
R4	14.6 ± 2.3	15.9 ± 1.6	17.0 ± 3.4	18.6 ± 7.2	33.5 ± 9.1	19.5 ± 0.9
L2	15.4 ± 5.1	26.6 ± 7.4	22.2 ± 2.2	17.5 ± 6.1	15.1 ± 7.6	16.4 ± 7.9
S2	21.7 ± 9.6	22.5 ± 11.8	26.7 ± 9.0	48.9 ± 25.5	23.5 ± 14.0	26.6 ± 9.2
S3	17.3 ± 6.8	20.3 ± 10.6	26.1 ± 6.0	18.9 ± 11.6	16.4 ± 11.0	21.1 ± 6.8
L3	11.5 ± 3.6	16.3 ± 4.9	21.0 ± 9.2	24.1 ± 18.0	16.2 ± 9.3	11.4 ± 1.9
R6	17.9 ± 4.1	22.6 ± 3.8	20.7 ± 2.2	31.7 ± 2.8	26.8 ± 11.4	18.3 ± 6.0

## Data Availability

The data presented in this study are available upon request from the corresponding author. The data are not publicly available due to privacy reasons.
